# Low-Molecular Weight BDNF Mimetic, Dimeric Dipeptide GSB-106, Reverses Depressive Symptoms in Mouse Chronic Social Defeat Stress

**DOI:** 10.3390/biom11020252

**Published:** 2021-02-10

**Authors:** Tatiana A. Gudasheva, Anna V. Tallerova, Armen G. Mezhlumyan, Tatyana A. Antipova, Ilya O. Logvinov, Yulia N. Firsova, Polina Y. Povarnina, Sergey B. Seredenin

**Affiliations:** 1 Department of Medicinal Chemistry, V.V. Zakusov Research Institute of Pharmacology, Baltijskaya 8, 125315 Moscow, Russia; antatatall@gmail.com (A.V.T.); armezhlumyan@gmail.com (A.G.M.); yulia.firsova@gmail.com (Y.N.F.); povarnina@gmail.com (P.Y.P.); 2 Department of Pharmacogenetics, V.V. Zakusov Research Institute of Pharmacology, Baltijskaya 8, 125315 Moscow, Russia; zenina_tatyana@mail.ru (T.A.A.); logvinov_ilya@mail.ru (I.O.L.); seredeninpharm@mail.ru (S.B.S.)

**Keywords:** BDNF, dipeptide mimetic, GSB-106, antidepressant-like activity, chronic social defeat stress

## Abstract

A mimetic of the BDNF loop 4, bis (N-monosuccinyl-L-seryl-L-lysine) hexamethylenediamide, named GSB-106, was designed and synthesized in our scientific group. The compound activated TrkB, MAPK/ERK, PI3K/AKT, and PLCγ in in vitro experiments. In vivo experiments with rodents revealed its antidepressant-like activity in the forced swim and the tail suspension tests at the dose range of 0.1–5.0 mg/kg (i.p., p.o.). However, GSB-106 was not studied in depression models modulating major depression in humans. In the present study, the GSB-106 antidepressant-like activity was revealed in mice at the depression model induced by 28-day social defeat stress with 21-days oral administration (0.1 mg/kg) after stress. At the same time, GSB-106 restored reduced locomotor activity and completely eliminated the anhedonia manifestations. The compound also restored reduced levels of synaptophysin and CREB in the hippocampus. In addition, the Trk receptor antagonist K252A, and the PLC inhibitor U73122, were found to completely block the antidepressant-like activity of GSB-106 in the forced swimming test in mice. Thus, the present results demonstrate the dipeptide BDNF mimetic GSB-106 reversed depressive-like behavior and restored hippocampal neuroplasticity in a rodent depression model. These effects of GSB-106 are probably regulated by TrkB signaling.

## 1. Introduction

According to modern conceptions, BDNF-TrkB signaling associated with the neuroplasticity maintenance plays a core role in the pathogenesis and treatment of depressive disorder [1]. BDNF is a key regulator of synaptogenesis, neuro/gliogenesis, and synaptic plasticity—the processes underlying neuroplasticity [2,3]. BDNF at intracerebral administration produces long-lasting antidepressant-like effects in experimental models of depression [4].

However, the clinical use of BDNF is limited due to its poor pharmacokinetic properties and undesirable side effects. Creation of low-molecular weight mimetics that would have the therapeutic effects of the full-length neurotrophin and would be devoid of its disadvantages seems to be the optimal way to overcome these problems. Low-molecular weight BDNF mimetics are being developed by a number of scientific groups [5,6].

A mimetic of the BDNF loop 4 GSB-106, being substituted dimeric dipeptide bis (N-monosuccinyl-L-seryl-L-lysine) hexamethylenediamide, was designed and synthesized at the V.V. Zakusov Research Institute of Pharmacology [7]. Previously GSB-106 was found to activate TrkB receptor and its main signal transduction pathways: MAPK/ERK, PI3K/AKT, and PLCγ [8,9]. GSB-106 antidepressant activity was revealed in the forced swimming test and tail suspension test in rats and mice at intraperitoneal (i.p.) (0.1–1.0 mg/kg) and oral (0.5–5.0 mg/kg) administration [10,11]. GSB-106 ability to stimulate hippocampal synapto- and neurogenesis was shown in experiments in mice [12,13]. However, GSB-106 has not been studied in the depression models modulating major depression in humans previously. Therefore the present study aim was to investigate the GSB-106 effects in chronic social defeat stress model in mice after three weeks of oral administration. Chronic social stress in mice is one of the most appropriate depression models, since it has good predictive validity and phenomenological and pathophysiological similarities with depressive disorder [14]. This model was used to study GSB-106 effects on sucrose preference and motor activity, as well as on the content of the synaptic marker synaptophysin and neuroplasticity marker CREB in the hippocampus. We chose the hippocampus as the main brain structure undergoing neurodegenerative changes in depression. The duration of GSB-106 administration corresponded to the full neurogenesis cycle duration.

TrkB receptor antagonist K252A and the PLCγ inhibitor U73122 effects on the GSB-106 antidepressant activity in the forced swim test were studied to confirm the BDNF-like action mechanism of the dipeptide.

## 2. Materials and Methods

### 2.1. Animals

Male C57BL/6 mice (10 weeks) weighing 19–25 g and male BALB/c mice (8 weeks) weighing 20–22 g were obtained from the Animal Breeding Facility Branch Stolbovaya of the Federal State Budget Institution of Science, the Scientific Center for Biomedical Technologies of the Federal Medical and Biological Agency, Russia. All animals were housed in a 12-h dark/light cycle, 22 °C ± 2 °C temperature and 60 ± 10% humidity. All the mice were put on a standard diet with food and water available ad libitum. The study was conducted complied with the requirements of Russian Federation Ministry of Health Order №199 of 1 April 2016 “On Approval of the Rules of Good Laboratory Practice” and GOST 33215-2014 “Guidelines for accommodation and care of animals. Environment, housing and management” (http://protect.gost.ru/document.aspx?control=7&id=202494 (accessed on 5 April 2019)) and Directive 2010/63/EU of the European Parliament and of the Council of 22 September 2010 “On the Protection of Animals Used for Scientific Purposes.” The animals were kept according the sanitary and epidemiological rules 2.2.1.3218-14 “On Sanitary and Epidemiological Requirements for the Design, Equipment, and Maintenance of Experimental Biological Clinics (Vivariums),” approved by the Russian Federation Chief State Sanitary Doctor Resolution №51 of 29 August 2014. All the experiments were approved by the Institutional Animal Care and Use Committee of V.V. Zakusov Research Institute of Pharmacology, Moscow (order number 5A of 26 April 2019).

### 2.2. Chemicals

GSB-106, bis (N-monosuccinyl-L-seryl-L-lysine) hexamethylenediamide, was synthesized in the Department of Medicinal Chemistry of the V.V. Zakusov Research Institute of Pharmacology as described previously [7]. The dosage form of GSB-106 for oral administration was developed and produced in the Experimental-technological department of the V.V. Zakusov Research Institute of Pharmacology (RF Patent 2697254. Priority date: 28 February 2018. Publ. 13 August 2019) and contained 1% of GSB-106 and 99% of a filler consisting of lactose, microcrystalline cellulose, polyethylene glycol-polyvinyl alcohol copolymer, and magnesium stearate.

Folin’s reagent was purchased from Merck KGaA (Darmstadt, Germany), bovine serum albumin (BSA), protease inhibitor cocktail, Na_3_VO_4_, K252A, 1-(6-((17-3-methoxyestra-1,3,5(10)-trien-17-yl) amino)hexyl)-1H-pyrrole-2,5-dione (U73122) were purchased from Sigma Aldrich Co., Ltd. (St. Louis, MO, USA). Sodium dodecyl sulfate (SDS), Tween-20, TEMED, bis-acrylamide, Tris, DTT-dithiothreitol, EDTA, Triton X-100, glycerin were purchased from Bio-Rad Laboratories (Hercules, CA, USA). Na-deoxycholate was purchased from AppliChem GmbH (Darmstadt, Germany). Primary polyclonal antibodies against synaptophysin were purchased from BD Biosciences (San Jose, CA, USA), against CREB, phospho-CREB was purchased from Cell Signaling Technology (Danvers, MA, USA), against beta-actin was purchased from Abcam (Cambridge, UK). Secondary antibodies anti-rabbit IgG conjugated with horseradish peroxidase were purchased from Abcam (Cambridge, UK). Amitriptyline was purchased from Moscow Endocrine Plant (Moscow, Russia).

### 2.3. Chronic Social Defeat Stress (CSDS) Procedure

Chronic social defeat stress in male C57Bl/6 mice was modeled according to the international protocols [14,15]. The experimental male mice were kept in pairs in cages (28 × 14 × 10 cm), divided in half by a perforated Plexiglas divider, one mouse per compartment. The divider was removed for 10 min daily, allowing the animals to have direct contact. One of the mice acted as an “aggressor”, and the second as a “victim”. In the case of overly aggressive attacks from the aggressor (biting continued even after the subordinate mouse demonstrated the submissive posture), the contact was terminated earlier then 10 min. Subordinate animals were exposed to stress every day for 28 d, which led to the development of severe depression in mice according to the literature data [16,17]. The victims were changed every day which created unpredictable conditions to victims in aggressor’s novel cages and led to additional stress. To the next experimental step all 28-days stressed mice were tested in IR-actimeter with locomotor activity registration. Then only mice with significantly decreased locomotor activity in comparison with non-stressed group were taken and equally randomized to the experimental treatment groups.

### 2.4. Drug Treatments

The subordinate mice were randomly divided into 2 groups, 8 animals in each: “CSDS”, “CSDS + GSB-106”. There were also 2 groups of animals, not exposed to stress, 10 mice in each group, which were kept for 28 d and then received placebo or GSB-106 dosage form in parallel with the animals exposed to stress. Control animals without stress (“Control” group) and animals in the “CSDS” group were orally administered with the dosage form vehicle suspended in 1% starch solution (placebo), once daily for 21 d. Dosage form of GSB-106 (tablets) was suspended in 1% starch solution and orally administered to mice from the groups “Control + GSB-106” and “CSDS + GSB-106” at a dose of 0.1 mg/kg (active pharmaceutical ingredient) daily, once, for 21 d. The GSB-106 dose was selected based on previous experiments [18]. The administration of drugs was started next day after 28-day social stress following the locomotion assessment. During the drug treatments, the mice were kept in groups of 4 animals per cage.

### 2.5. Locomotion

An infrared actimeter (Panlab, Barcelona, Spain) with ActiTrack software was used to register locomotor activity. Motor activity of the mice was recorded for 3 min. The test was performed 24 h after the end of 28-day social stress and then 24 h after the last drug administration.

### 2.6. Sucrose Preference Test

After the motor activity rating, the mice, seated individually, were given 24-h free access simultaneously to two bottles, one of which contained a 1% sucrose solution, and the other—pure water. Positions of the bottles were switched after the half of the time during the test. Water and sucrose solutions consumption was assessed by weighing the bottles before and after the test. The sucrose solution preference was calculated by following formula: (M suc)/(M suc + M water)*100%, where M suc—consumed sucrose solution mass, M water—consumed water mass.

Decrease in sucrose solution preference below the control group was regarded as the anhedonia development [19].

### 2.7. Sample Collection

Five days after the last sucrose preference test, the mice were decapitated. The hippocampus was collected into liquid nitrogen and stored at −80°C until protein detection.

**Figure 1:**
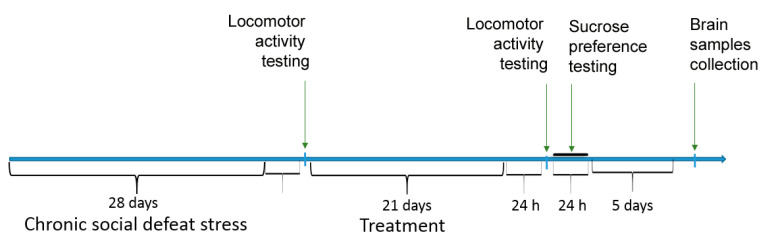
The design of the experiment the low-molecular-weight BDNF mimetic GSB-106 effects in a model of chronic social stress in mice.

The design of the experiment is shown in Figure 1.

### 2.8. Western Blotting

Brain tissue samples were homogenized in lysis buffer (50 mM Tris-HCl, 5mM EDTA, 1mM DTT, 1% Triton X-100) pH 7.5 at a ratio of 1:10 (tissue:buffer), at 4 °C for 5 min, then placed into a 1.5 mL centrifuge tube and centrifuged at 13,000 rpm for 10 min at 4 °C. The cytosolic fraction (supernatant) was used for determination of synatophysin, the precipitate was used for isolation of the nuclear fraction and determination of CREB and phospho-CREB.

The precipitate was lysed in a buffer (50 mM Tris-HCl, 150 mM NaCl, 1mM EDTA, 1mM EGTA, 1mM DTT, 1% Triton X-100, 0.5% Na-deoxycholate, 0.1% SDS, 10% glycerol, 1 mM Na_3_VO_4_) pH 7.8 at a ratio of 4:6 (tissue:buffer) for 30 min at 0 °C, stirring every 10 min. Then the samples were centrifuged at 14,000 rpm for 30 min at 4 °C. The supernatant was collected. The protein concentration in the samples was measured by the Folin-Lowry method. Proteins were separated in 12% polyacrylamide gel (PAGE). The transfer of proteins from PAGE to the PVDF membrane was carried out by electroelution for 45 min.

Western blots were preincubated in TBS-T buffer (supplemented with 1% Tween-20) with 3% (*w*/*v*) BSA for 1 h. Then the membranes were incubated with primary antibodies against synaptophysin at a dilution of 1:500, against CREB at a dilution of 1:500, against phospho-CREB at a dilution of 1:1000, against beta-actin at a dilution of 1:5000 at 4 °C overnight. The membranes were washed with TBS-T buffer (supplemented with 1% Tween-20) with 0.5% (*w*/*v*) BSA and then were incubated with secondary antibodies conjugated with horseradish peroxidase (dilution 1:500) within 45 min. Proteins were detected after wash from secondary antibodies in TBS-T buffer (supplemented with 1% Tween-20) with 0.5% (*w*/*v*) BSA in reaction with ECL reagents using a gel documenting system Alliance UVITEC (UK). Densitometry of the obtained images was performed using the program GIMP2. Beta-actin was used as a loading control.

### 2.9. Pharmacological Inhibitory Analysis

Forced swimming test [20] was used to study the mechanism of GSB-106 antidepressant-like action. The test was carried out as described previously [11,21]. The apparatus setup consisted of 5 transparent plastic cylinders 10 cm in diameter and 30 cm in height, separated by black plastic opaque dividers to prevent visual contact of animals during the study. The cylinders were filled with water 22 °C temperature by 2/3. On the first day, mice were placed in cylinders with water for 10 min. After 22 h, the animals were placed again for 5 min under the same conditions. The video recording of the experiment was conducted. The video was processed using the ANY-maze software (Ireland). The total time of animal immobility was registered.

The studies of the contribution of TrkB and PLCγ activation by the GSB-106 antidepressant activity were conducted with the Trk receptor blocker K252A and the specific inhibitor of phospholipase C U73122. K252A and U73122 were dissolved in 1% DMSO/physiological saline solution and injected i.p. 30 min after the first swimming session at 25 μg/kg [22] and 30 mg/kg [23] doses, respectively. GSB-106 was dissolved in distilled water and injected at 0.1 mg/ kg dose i.p., 30 min after the inhibitors administration. A tricyclic antidepressant amitriptyline was used as a reference drug in the physiological saline and injected i.p. at 10 mg/kg dose [24].

### 2.10. Statistical Analysis

Intergroup differences were assessed by Student’s t-test or by the Mann–Whitney U test in case of 2 groups comparison or by two-way ANOVA followed by Tukey’s test or LSD test when more than 2 groups compared. Differences were considered statistically significant at *p* ≤ 0.05. Data were presented as means and standard errors of the mean.

## 3. Results

### 3.1. GSB-106 Restores Decreased Locomotion in CSDS Mice

The motor activity in mice one day after the CSDS was found to be significantly reduced by 1.4 times (F (23, 9) = 2.49; *p* = 0.0002) compared with animals that were not subjected to stress (Table 1).

Reduced locomotor activity in stressed animals receiving placebo was also observed 22 d after the end the stress (Table 2). Two-way ANOVA analysis of locomotion data showed statistically significant CSDS effect (F (1, 30) = 5.278, *p* = 0.0287). GSB-106 effect and factors interaction were not significant (F (1, 30) = 0.6213, *p* = 0.4368; F (1, 30) = 1.98, *p* = 0.1697). Post-hoc Fisher’s LSD test revealed statistically significant differences between “Control” and “CSDS” groups (*p* = 0.016) and the tendency (*p* = 0.14) for GSB-106 to in-crease locomotion in CSDS mice. However, significant differences both between “Control” and “CSDS” groups (*p* = 0.014) and between “CSDS” and “CSDS + GSB-106” groups (*p* = 0.05) were established using Mann–Whitney U-test.

### 3.2. GSB-106 Completely Restores Sucrose Solution Preference Impaired in CSDS Mice

Two-way ANOVA showed that the interaction (F(1, 24) = 10.33, *p* = 0.0037) between CSDS and GSB-106 treatment was significant. Post hoc test revealed that stressed animals treated with placebo showed a statistically significant (*p* < 0.012) reduction in sucrose solution preference by 20% comparing with non-stressed control animals received placebo. GSB-106 administration promoted complete recovery of sucrose solution preference to the control level (*p* = 0.0039) (Table 3). Chronic GSB-106 administration to non-stressed animals did not affect this parameter.

### 3.3. GSB-106 Restores Reduced Immunoreactivity to Synaptophysin in the Hippocampus of CSDS Mice

**Figure 2:**
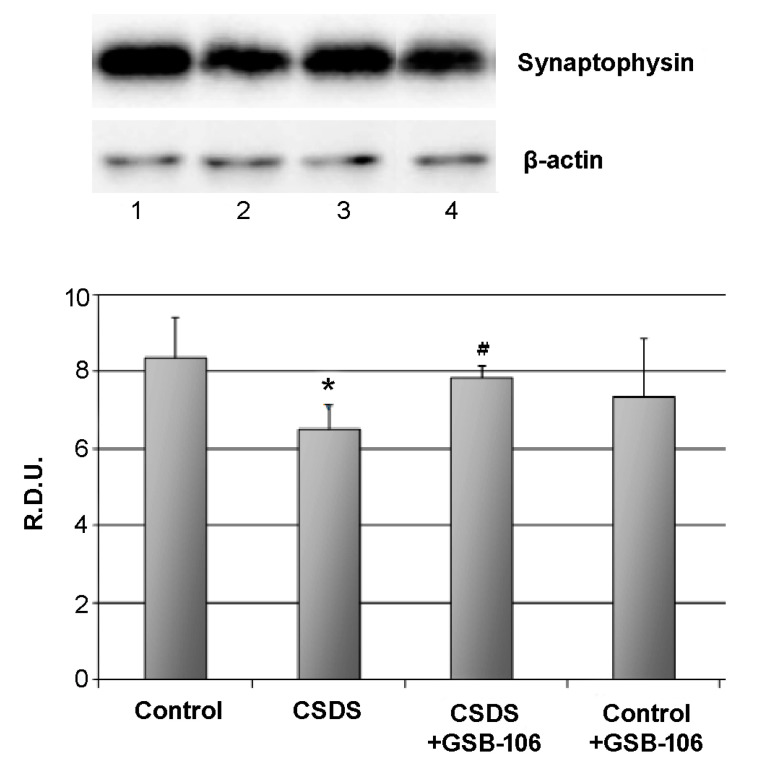
Dipeptide GSB-106 restores decreased synaptophysin level in hippocampus of CSDS mice.Western blot analysis data. R.D.U—relative densitometric units. Lanes: 1—Control, 2—CSDS, 3—CSDS + GSB-106, 4—Control + GSB-106.Data are presented as means and standard errors of the mean. *—*p* < 0.05 as compared to the “Control “ group, #—*p* < 0.05 as compared to the “CSDS” group (Mann–Whitney U test). There was no statistically significant difference between the β-actin bands.

In CSDS mice a statistically significant synaptophysin level decrease was observed (8.4 ± 1.0 R.D.U. in the control; 6.5 ± 0.6 R.D.U. in the “CSDS” group). Treatment with GSB-106 statistically significantly (*p* < 0.05) restored the reduced synaptophysin level (up to 7.9 ± 0.3 R.D.U.) (Figure 2). GSB-106 therapeutic effect was 74%. Administration of GSB-106 to control animals did not affect the synaptophysin rate (Figure 2).

### 3.4. GSB-106 Dipeptide Completely Restores Reduced Immunoreactivity to CREB and Phospho-CREB in the Hippocampus of CSDS Mice 

**Figure 3:**
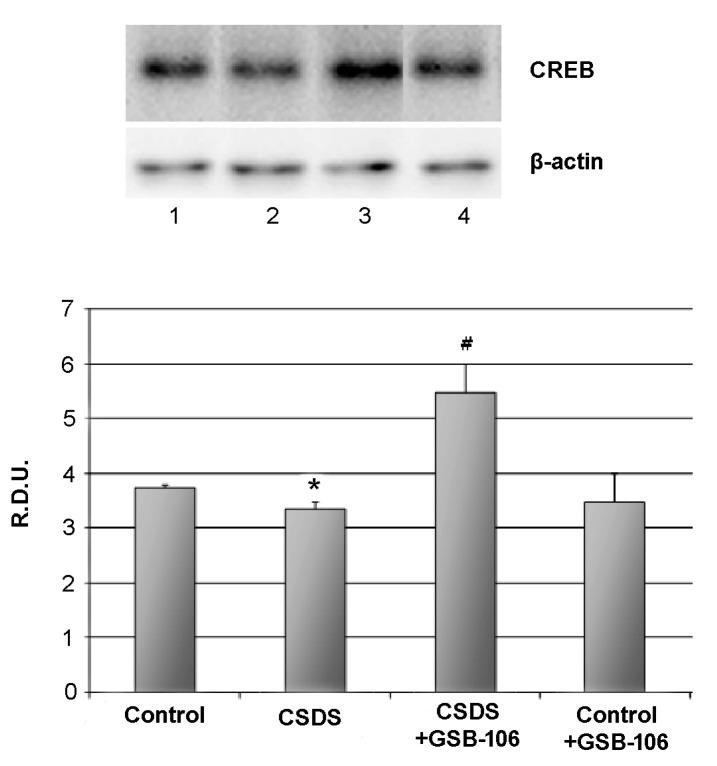
Dipeptide GSB-106 completely restores decreased CREB level in hippocampus of CSDS mice.Western blot analysis data. R.D.U—relative densitometric units. Lanes: 1—Control, 2—CSDS, 3—CSDS + GSB-106, 4—Control + GSB-106. Data are presented as means and standard errors of the mean. *—*p* < 0.05 as compared to the “Control “ group, #—*p* < 0.05 as compared to the “CSDS” group (Mann–Whitney U test). There was no statistically significant difference between the β-actin bands.

The level of the transcriptional nuclear factor CREB also statistically significantly (*p* < 0.05) decreased in response to social stress up 3.8 ± 0.1 R.D.U in the control to 3.4 ± 0.2 R.D.U in the “CSDS” group. Treatment with GSB-106 restored (*p* < 0.05) the reduced CREB level, and the CREB rate was even higher than in the control (5.5 ± 0.5 R.D.U) (Figure 3). GSB-106 administration to control animals did not affect the CREB level (Figure 3).

**Figure 4:**
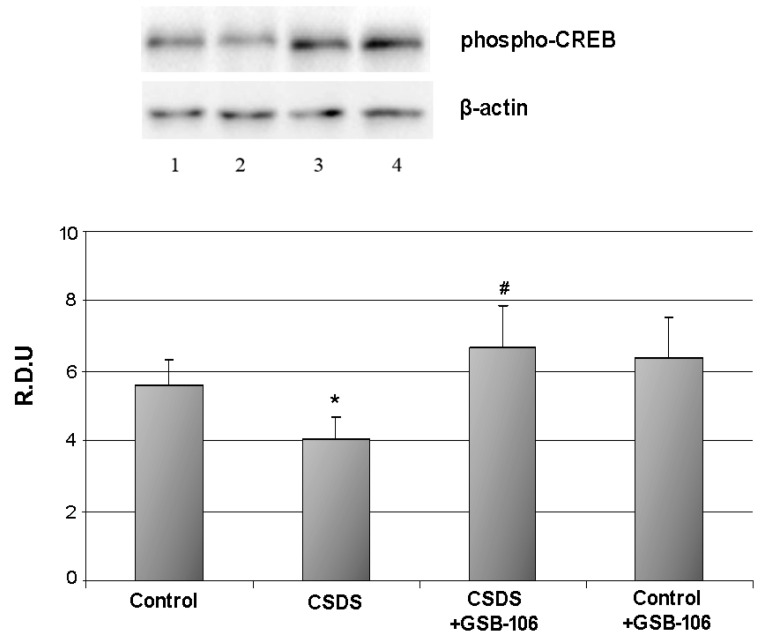
Dipeptide GSB-106 completely restores decreased pCREB level in hippocampus of CSDS mice.Western blot analysis data. R.D.U—relative densitometric units. Lanes: 1—Control, 2—CSDS, 3—CSDS + GSB-106, 4—Control + GSB-106. Data are presented as means and standard errors of the mean. *—*p* < 0.05 as compared to the “Control “ group, #—*p* < 0.05 as compared to the “CSDS” group (Mann–Whitney U test). There was no statistically significant difference between the β-actin bands.

The level of the phospho-CREB was statistically significantly (*p* < 0.05) decreased in response to social stress up 5.6 ± 0.7 R.D.U in the control to 4.1 ± 0.6 R.D.U in the “CSDS” group. Treatment with GSB-106 restored (*p* < 0.05) the reduced phospho-CREB level, and the phospho-CREB rate was even higher than in the control (6.7 ± 1.2 R.D.U) (Figure 4). GSB-106 administration to control animals did not affect the phospho-CREB level (Figure 4).

### 3.5. TrkB Receptor Antagonist K252A Completely Blocks Antidepressant-Like Activity of GSB-106 Dipeptide in a Forced Swim Test in Mice

**Figure 5:**
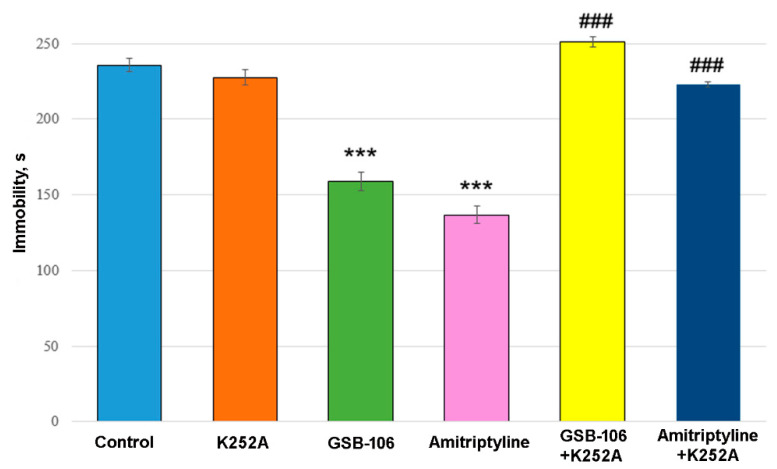
The antagonist of TrkB receptors K252A completely blocks the antidepressant-like activity of the GSB-106 dipeptide and Amitriptyline in the forced swimming test in mice. Data are presented as means and standard errors of the mean. *** *p* < 0.0001 statistical significance of differences compared to the “Control” group; ### *p* < 0.0001 statistical significance of differences compared to the “GSB-106” or “Amitriptyline” group (two-way ANOVA with Tukey’s post hoc test).

Two-way ANOVA showed a significant treatment effect [F(2, 54) = 60.16, *p* < 0.0001], K252A effect [F(1, 54) = 215.3, *p* < 0.0001], and their interaction effect [F(2, 54) = 70.94, *p* < 0.0001] on immobility time. Post hoc test revealed that the dipeptide mimetic BDNF GSB-106 significantly (*p* < 0.0001) reduced the immobility time in the forced swimming test in mice by 32% compared to the control (Figure 5). Amitriptyline also statistically significantly (*p* < 0.0001) reduced the immobility time by 42.0% compared to the control group (Figure 5).

Administration of K252A completely abolished the antidepressant effects of both GSB-106 and Amitriptyline (Figure 4). At the same time, the K252A did not affect the immobility time by itself.

Obtained results indicate that the antidepressant-like action of GSB-106 and Amitriptyline is attributed to the TrkB receptor activation.

### 3.6. PLC Inhibitor U73122 Completely Blocks Antidepressant-Like Activity of GSB-106 Dipeptide in the Forced Swimming Test in Mice

**Figure 6:**
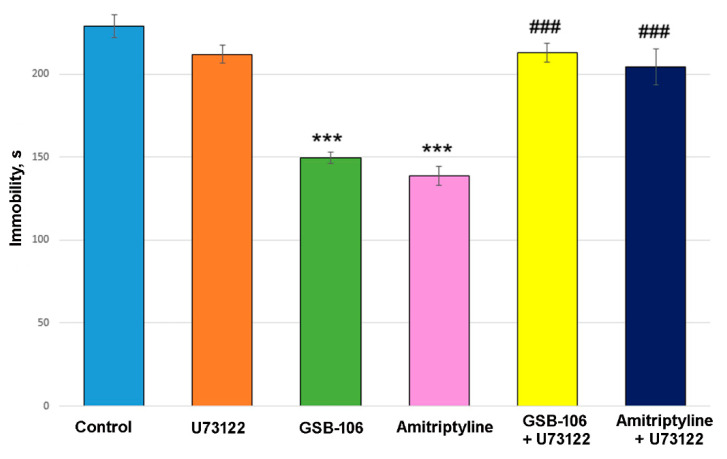
The PLC inhibitor U73122 blocks the antidepressant-like activity of the GSB-106 dipeptide and Amitriptyline in the forced swimming test in mice. *** *p* < 0.0001 statistical significance of differences compared to the “Control” group; ### *p* < 0.0001 statistical significance of differences compared to the “GSB-106” or “Amitriptyline” group (two-way ANOVA with Tukey’s post hoc test).

Two-way ANOVA showed a significant treatment effect (F(2, 54) = 29.52, *p* < 0.0001), U73122 effect (F(1, 54) = 46.35, *p* < 0.0001), and their interaction effect (F(2, 54) = 24.32, *p* < 0.0001) on immobility time. Post hoc test revealed that the dipeptide BDNF mimetic GSB-106 significantly (*p* < 0.0001) reduced the immobility time in the forced swimming test in mice by 35% compared to the control group (Figure 6). Amitriptyline also statistically significantly (*p* < 0.0001) reduced the immobility time by 39% compared to the control group (Figure 6). Administration of U73122 almost completely abolished the antidepressant-like effects of both GSB-106 and Amitriptyline (Figure 6). The inhibitor did not affect the time of immobility by itself.

## 4. Discussion

In the present study, the recovery of behavioral and biochemical parameters of a depressive-like state in mice subjected to chronic social stress at chronic oral administration of the dipeptide BDNF mimetic GSB-106 was demonstrated for the first time.

The depressive-like behavior in CSDS mice was assessed by the anhedonia and motor retardation development, which are considered as the main depression symptoms [25].

In the sucrose preference test, the most common method for evaluation anhedonia in rodents [26], the preference for sucrose solution consumption was found to be reduced by 20% in the mice exposed to chronic stress compared to control animals. In addition, chronic social stress led to a 1.4-fold decrease in motor activity compared to non-stressed animals. The deficit in locomotion in stressed animals persisted for at least three weeks after the end of stress. These data are consistent with the literature data on the motor activity decrease in mice in a chronic social defeat stress model of depression [27]. 

Biochemical studies of the hippocampal tissue showed that stressed mice had a statistically significantly reduced immunoreactivity to the synaptic marker synaptophysin (by 20%) and transcription factor CREB (by 10%). Synaptophysin is a protein of presynaptic vesicles regulating their endocytosis [28]. The severity of depressive disorder is known to correlate inversely with the synapses density in the prefrontal cortex and hippocampus [29], while the synapses density correlates with the synaptophysin level [30]. CREB is a downstream component in a number of signaling cascades, including BDNF/TrkB/MAPK/ERK [31] and regulates the transcription of many genes involved in the brain in such functions as neuronal survival, synaptogenesis, neurogenesis, synaptic plasticity, learning, and memory [32,33,34]. Among others, CREB stimulates transcription of the BDNF gene [35]. CREB is considered a neuroplasticity [36]. As in the case of BDNF, the level of CREB in the hippocampus and cortex decreases with depression and returns to normal after antidepressant therapy [31,37]. 

In the present study, the BDNF mimetic dipeptide GSB-106 was found to correct depressive-like behavior at chronic administration, promoting the restoration of motor activity with a therapeutic effect of 60% and a complete recovery of anhedonia manifestations in mice subjected to chronic 28-day social stress. In addition, GSB-106 promoted the restoration of the synaptophysin level with a therapeutic effect of 74% and the complete restoration of the CREB level in the hippocampus of CSDS mice. Based on the literature data, it can be suggested that the antidepressant-like effects of GSB-106 are associated with the restoration of impaired synaptogenesis (synaptophysin) and neuroplasticity (synaptophysin and CREB).

Previously, by Western blot analysis it was shown that GSB-106 activates TrkB receptors and their main signal transduction pathways: MAPK/ERK, PI3K/AKT, and PLCγ. [8,9].

A large amount of experimental and clinical data indicates that the antidepressant activity of BDNF is mediated by its interaction with TrkB receptors [2]. In the present study, the Trk receptor blocker K252A effect on the antidepressant activity of the dipeptide was investigated in the forced swimming test in mice to confirm the BDNF-like mechanism of GSB-106 action. K252A completely abolished the antidepressant-like activity of both GSB-106 and Amitriptyline.

The PLCγ postreceptor signaling cascade is one of the CREB activators [31]. The effect of the PLC inhibitor U73122 on the dipeptide effects in the forced swimming test in mice was considered to reveal the contribution of the cascade to the antidepressant activity of GSB-106. U73122 was found to completely abolished the antidepressant-like effects of GSB-106.

## 5. Conclusions

Thus, the present results demonstrate the dipeptide BDNF mimetic GSB-106 reversed depressive-like behavior and restored hippocampal neuroplasticity in a rodent depression model. These effects of GSB-106 are probably regulated by TrkB signaling.

## Figures and Tables

**Table 1:** Effect of Chronic social defeat stress (CSDS) on locomotor activity in mice.

Group	n	Motor Activity
Control	20	620.0 ± 24.4
CSDS	16	440.8 ± 24.9 *

Locomotion was assessed in an infrared actimeter 24 h after the end of stress.; *—*p* < 0.01—statistical significance of differences compared with the control group according to the unpaired Student’s *t*-test.

**Table 2:** Effect of dipeptide GSB-106 on locomotion in CSDS mice.

Group	n	Motor Activity
Control	10	493.8 ± 39.7
Control + GSB-106	10	471.2 ± 35.5
CSDS	8	358.6 ± 32.5 *
CSDS + GSB-106	8	438.8 ± 31.2 #

Locomotion was assessed in an infrared actimeter 24 h after the end of drugs administration.; * *p* < 0.05—statistical significance of differences compared with the “Control” group; # *p* ≤ 0.05—statistical significance of the differences compared with the “CSDS” group (Mann–Whitney U test).

**Table 3:** Effect of dipeptide GSB-106 on sucrose preference in CSDS mice.

Groups	n	Preference of 1% Sucrose Solution, %
Control	10	67.3 ± 3.6
Control + GSB-106	10	61.3 ± 3.4
CSDS	8	53.7 ± 3.9 *
CSDS + GSB-106	8	69.7 ± 3.6 #

* *p* < 0.05—statistical significance of differences compared with the “Control” group; # *p* < 0.05—statistical significance of the differences compared with the “CSDS group; (two-way ANOVA with LSD post hoc test).

## Data Availability

The datasets during and/or analysed during the current study available from the corresponding author on reasonable request.
